# A decisive technical leap forward for personalized medicine to treat mitochondrial diseases

**DOI:** 10.1038/s44321-025-00232-4

**Published:** 2025-04-09

**Authors:** Abi S Ghifari, Martin Ott

**Affiliations:** Department of Medical Biochemistry and Cell Biology, Institute of Biomedicine, University of Gothenburg, 405 30 Gothenburg, Sweden

**Keywords:** Genetics, Gene Therapy & Genetic Disease, Organelles

## Abstract

M. Ott and A. Ghifari discuss a method to decrease heteroplasmy levels of disease-linked mtDNA in cardiac and skeletal muscles as a potential strategy for personalized medicine to treat mitochondrial diseases as reported by M. Minczuk and colleagues, in this issue of *EMBO Mol Med*.

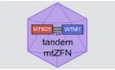

## Teaser

Mitochondrial diseases, arising from mutations in mitochondrial DNA (mtDNA), are a diverse group of disorders that lead to severe conditions. Expression of mtDNA is a prerequisite for ATP production by oxidative phosphorylation. Individual cells contain many hundred copies of mtDNA and mutated mtDNA coexist with wild type genomes, a phenomenon termed heteroplasmy, rendering the diagnosis and treatment of mtDNA-caused diseases challenging. In this issue of *EMBO Molecular Medicine*, Nash et al report an effective method to decrease heteroplasmy levels of disease-linked mtDNA in cardiac and skeletal muscles of a mouse model. For this, the team developed a novel strategy relying on a tandem mitochondrial-targeted zinc finger nuclease (tandem mtZFN) delivered via adeno-associated virus (AAV) particle to tissues. This approach significantly reduced the mtDNA mutation load, thereby shifting heteroplasmy levels and alleviating molecular phenotypes associated with the mutation. This breakthrough provides a promising new avenue for therapeutic interventions to treat mitochondrial diseases.

Mitochondria are the powerhouses of our cells, responsible for generating most cellular ATP through the oxidative phosphorylation (OXPHOS) pathway. In addition to providing cellular ATP, mitochondria are also essential for cellular homeostasis and various metabolic pathways, including amino acid metabolism, reactive oxygen species (ROS) and calcium signaling, biosynthesis of heme and iron-sulfur clusters, and cell death. The maternally inherited (Gupta et al, 2023) mitochondrial DNA (mtDNA) is a small, double-stranded circular genome that encodes core OXPHOS proteins and translation machinery components, including ribosomal RNAs (rRNAs) and transfer RNAs (tRNAs). These components are necessary for mitochondrial functionality, primarily cellular respiration. Mutations in any of these components may lead to health disorders, most notably mitochondrial diseases.

**Figure 1:**
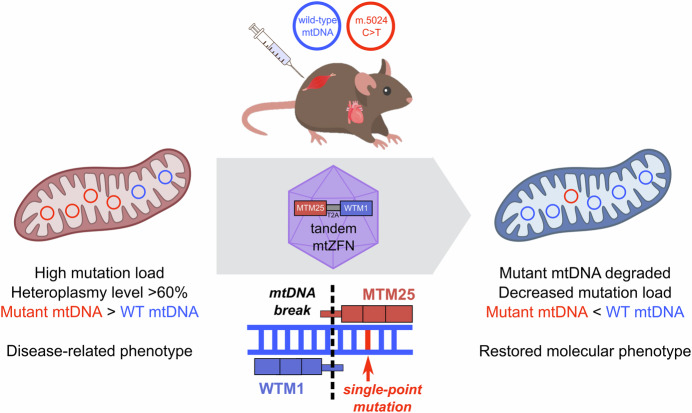
Treatment of disease-associated mitochondrial heteroplasmy with a novel tandem mitochondria-targeted zinc-finger nuclease (mtZFN) technology. The target is a mouse model harboring a high level of heteroplasmy or higher population of disease-related mutated mitochondrial DNA (mtDNA) m.5024 C > T (red) compared to wild-type (WT) mtDNA (blue). In contrast to the classical approach of using separate mtZFN monomers, this study used a tandem mtZFN dimer comprised of a mutation-specific monomer MTM25 and a wild-type binding monomer WTM1 linked with a translation-skipping T2A sequence. The recombinant construct was delivered via adeno-associated virus (AAV) capsid to cardiac and skeletal muscles. Upon introduction of the tandem mtZFN sequence, both monomers recombine on the mtDNA containing the target single-point mutation. The nuclease tails of both monomers then form an active dimer to cleave the double strand, leading to its degradation and, subsequently, to its replacement by healthy wild-type mtDNA. The study thus reported a significant decrease in heteroplasmy load and a partial alleviation of the molecular phenotype associated with the mutation.

Mitochondrial diseases are a group of human diseases, which are among the most commonly inherited disorders in adults, with at least 1 in 4300 of the population being affected (Gorman et al, 2015). These diseases are largely associated with mutations in mtDNA, in most cases a single point mutation. Mutation rates in mtDNA are much higher compared to the nuclear DNA due to a poor DNA repair system and a constant exposure to ROS, particularly in higher energy-demanding tissues such as the brain, eyes, heart, and muscles (Gorman et al, 2016). Mutated mtDNA typically co-exists with wild-type mtDNA molecules, a condition called heteroplasmy (Fig. 1). The severity of diseases typically correlates with the proportion of mutant versus wild-type mtDNA, where these heteroplasmy levels within a cell often determine the phenotypic outcomes. Consequently, most mitochondrial diseases are characterized by a heteroplasmy effect, where disease symptoms become evident when heteroplasmy level exceeded a >60% threshold (Gammage et al, 2018).

Because of the central role of heteroplasmy in the manifestation of mitochondrial diseases, it is important to develop genetic strategies to reduce the amounts of mutated DNA in cells. A specific challenge is to target only the mutated mtDNA, while the wild-type mtDNA should be spared, thus requiring a highly specific recognition of the mutated form. Moreover, mtDNA, being located in the mitochondrial matrix, is enclosed by two biological membranes, which complicates the targeting of mtDNA-modifying strategies. For example, despite recent significant advances in CRISPR-Cas9 gene editing technologies, this technique failed to manipulate mammalian mtDNA due to the inability of mitochondria to import the guide RNA molecules necessary for DNA editing (Gammage et al, 2018). One strategy that has been developed to shift heteroplasmy was by targeted degradation of the mutated mtDNA via mitochondria-targeted nucleases (Gammage et al, 2018; Bacman et al, 2018; Zekonyte et al, 2021). By introducing a selective double-strand break specifically at the mutated site, the mutant mtDNA is rapidly degraded because mammalian mitochondria lack an efficient process to repair these lesions (Peeva et al, 2018). As the cellular copy numbers of mtDNA are maintained by the combination of expression of the mtDNA packaging factor called mitochondrial transcription factor A (TFAM) and through new rounds of replication, degraded mutant mtDNA will be replaced by a healthy copy of mtDNA, thereby shifting the heteroplasmy level (Gammage et al, 2018).

Previously, an approach employing mitochondria-targeted zinc-finger nuclease (mtZFN) that specifically targets mtDNA with a single point mutation has been demonstrated to efficiently reduce heteroplasmy level and rescue the associated molecular phenotypes (Gammage et al, 2018). This approach used a construct that consists of two mtZFN monomers that are expressed separately since its endonuclease tail requires two copies of each monomer to recognize and break the DNA double strand. The construct was designed to target mtDNA in a well-established mouse model bearing a heteroplasmic m.5024 C > T mutation, which destabilizes the structure of the mitochondrial alanyl-tRNA (tRNA^Ala^) required for mitochondrial protein synthesis. One monomer contains a zinc-finger domain that specifically binds to the mtDNA site containing the single-point mutation, which was referred to as mutant-specific monomer MTM25. The other monomer, called wild-type monomer WTM1, binds to the complementary sequence adjacent to the mutation site. When recombined on the mutated mtDNA in situ, both endonuclease monomers form an active dimer that cleaves the DNA double-strand (Gammage et al, 2018). The cleaved mtDNA will then get degraded, allowing more wild-type copies of mtDNA to be replicated to reach a new steady-state level of heteroplasmy.

Delivery of these split nucleases to mitochondria in often post-mitotic cells in tissue employs AAV capsids. However, these viruses have a limited capacity to load DNA and at the same time, when injected into the mouse, can provoke an immune response as an unwanted side effect. Hence, despite that this molecular strategy works so far in vivo, mtZFN MTM25 and WTM1 monomers need to be administered separately in two different AAV capsids, posing a challenge to translate this technology to humans. Because the two monomers need to bind to a specific mutation site, recombine, and form a dimer, both proteins should be available at the target location at roughly the same time, complicating dosage and injection timing. Additionally, separate administrations of AAV capsids lower the chance of recombination as well as triggering excessive immune responses against the virion particle, thereby posing a safety risk (Gammage et al, 2018). From the production perspective, separate injections also double the dosage amount and require an increased manufacturing capacity.

How can these technical constraints be tackled to improve the safety and efficacy of this technology to treat human patients? In a study published in this issue of *EMBO Molecular Medicine*, Nash et al (2025) reported a new strategy to incorporate a tandem mtZFN construct within a single AAV virion in contrast to the initial strategy to administer the mtZFN dimer separately. Compared to other mtDNA-modifying nucleases, mtZFNs provide a practical advantage due to their relatively small size. This opens the possibility to encode both mtZFN monomers within a single AAV genome. However, space restrictions still require that both monomers are coded within a joint open reading frame, which is then separated eventually to form two independent proteins. In this study, the authors made use of a viral T2A sequence inserted between the coding sequences of MTM25 and WTM1 monomers. This T2A sequence promotes the skipping of ribosomal peptide bond formation at a specific sequence during translation, upon which translation resumes. This leads to the production of two separate protein products from one open reading frame. Hence, when the AAV virion is successfully delivered to the target cell, both monomers are synthesized at the same time and imported into mitochondria, where they can recombine on mutant mtDNA to induce cleavage and, eventually, degradation (Fig. 1).

Using this tandem construct, the authors then demonstrated a substantial mtDNA heteroplasmy shift in both cardiac and skeletal muscles of the mouse model bearing 46–78% heteroplasmy m.5024 C > T mtDNA load. The tandem mtZFN construct was also slightly more effective in reducing the overall mutation load (~40% reduction) compared to mtZFN monomers administered in a separate AAV delivery (~28%). Since the mouse model m.5024 C > T has a disease-related phenotype of reduced level of tRNA^Ala^, this feature was then used to assess the restoration of phenotype. The steady-state level of tRNA^Ala^ was significantly improved in skeletal muscles upon mtZFN treatment. Taken together, these results demonstrated that this novel tandem mtZFN administration significantly reduces mtDNA heteroplasmy in both cardiac and skeletal muscles and is able to correct the disease-related phenotype more effectively compared to previously reported separate mtZFN treatment.

To assess the safety of tandem mtZFN delivery, various inflammatory and immune response markers were also determined. Following the tandem mtZFN administration, no significant changes of six markers were observed in the treated tissue compared to the mock control, indicating the absence of innate inflammatory or immune responses upon tandem mtZFN introduction. On the other hand, the administration of mtZFN on separate capsids significantly induced an immune response, especially at a higher dose. Overall, these analyses demonstrate that treatment of mtDNA heteroplasmy using tandem mtZFN had no significant adverse effect.

This novel technique provides an advantage over other AAV-delivered nuclease technologies since the entire heteroplasmy-modifying apparatus can be delivered within a single AAV virion. Therefore, it eliminates the need for co-infection of separate virions, reducing dosing requirements and potentially adverse immune responses. These factors are essential to translate this technology for clinical trials and future treatment of mitochondrial diseases in human patients. It is also interesting to extend this approach to treat other heteroplasmy-associated mitochondrial diseases like Leigh syndrome, Leber’s hereditary optic neuropathy (LHON), and Kearns-Sayre syndrome (KSS) (Gorman et al, 2016; Lake et al, 2016). Nevertheless, this successful trial of tandem mtZFN treatment represents a critical step toward the development of potential therapies for mitochondrial diseases. If successfully translated to humans, this approach could provide a means to personalize heteroplasmy-shifting treatment in patients, offering the possibility of restoring normal mitochondrial function, and alleviating the disease symptoms.
